# Ferumoxytol-Enhanced Cardiac Magnetic Resonance Angiography and 4D Flow: Safety and Utility in Pediatric and Adult Congenital Heart Disease

**DOI:** 10.3390/children9121810

**Published:** 2022-11-24

**Authors:** Pierangelo Renella, Jennifer Li, Ashley E. Prosper, J. Paul Finn, Kim-Lien Nguyen

**Affiliations:** 1 Diagnostic Cardiovascular Imaging Laboratory, Department of Radiological Sciences, 300 Medical Plaza, B119, Los Angeles, CA 90095, USA; 2 Department of Pediatric Cardiology, David Geffen School of Medicine, University of California, Los Angeles, CA 90055, USA; 3 Division of Cardiology, David Geffen School of Medicine, University of California, VA Greater Los Angeles Healthcare System, Los Angeles, CA 90055, USA

**Keywords:** ferumoxytol, ultrasmall superparamagnetic iron oxide nanoparticles (USPIO), gadolinium, contrast media, cardiac magnetic resonance, angiography, venography, congenital heart disease

## Abstract

Cardiac magnetic resonance imaging and angiography have a crucial role in the diagnostic evaluation and follow up of pediatric and adult patients with congenital heart disease. Although much of the information required of advanced imaging studies can be provided by standard gadolinium-enhanced magnetic resonance imaging, the limitations of precise bolus timing, long scan duration, complex imaging protocols, and the need to image small structures limit more widespread use of this modality. Recent experience with off-label diagnostic use of ferumoxytol has helped to mitigate some of these barriers. Approved by the U.S. FDA for intravenous treatment of anemia, ferumoxytol is an ultrasmall superparamagnetic iron oxide nanoparticle that has a long blood pool residence time and high relaxivity. Once metabolized by macrophages, the iron core is incorporated into the reticuloendothelial system. In this work, we aim to summarize the evolution of ferumoxytol-enhanced cardiovascular magnetic resonance imaging and angiography and highlight its many applications for congenital heart disease.

## 1. Introduction

Since the turn of the millennium, the field of cardiovascular disease has greatly benefitted from advances in cardiovascular magnetic resonance imaging and angiography (CMR/MRA). The high spatial and temporal resolution of CMR/MRA has given clinicians a reference standard for evaluating anatomy, morphology, function, and relationships between intracardiac anatomy and extracardiac vasculature [1,2]. Advances in methods aimed at myocardial tissue characterization, in the form of late gadolinium enhancement, multiparametric mapping techniques (T1, T2, T2*), and 4D flow have further added insight to disease processes, prognosis, and response to treatments in ischemic and non-ischemic cardiac pathologies as well as vascular diseases [3,4,5].

These innovations in CMR/MRA have gradually enhanced the clinical practice of pediatric and adult congenital heart disease (CHD) to the point that CMR/MRA is now accepted as an important diagnostic modality in the CHD diagnostic armamentarium [6,7]. The main advantage of CMR/MRA with respect to evaluation of complex cardiovascular anatomy and function is its inherent large field of view, volumetric acquisition, and absence of ionizing radiation. These attributes are valuable, particularly when repeated imaging throughout a CHD patient’s lifetime is needed, and during pre-procedural or pre-operative planning.

CMR/MRA is a 3D cross-sectional technique with a large field of view and clear extracardiac anatomic reference landmarks including the sternum, spine, and airways. Thus, CMR/MRA allows for the “on axis” display of ECG-gated dynamic (“cine”) images and can lead to more accurate definition of complex anatomy and measurement of cardiovascular structures [8,9]. Beyond anatomic and functional assessment, phase contrast MRI also allows for physiologic assessment of arterial and venous blood flow, intracardiac flow patterns, flow across heart valves, and shunt quantification [10]. As CMR/MRA protocols for CHD are often lengthy with the requirement for multiple breath-holds, younger patients, and some older children and adult patients, may require the use of sedation or general anesthesia [11]. However, the many advantages of comprehensive cardiovascular anatomic and physiological assessment, and the absence of ionizing radiation exposure, have made this modality indispensable in the field of pediatric and adult CHD [7,12].

In this review, we aim to summarize the available data on ferumoxytol-enhanced CMR/MRA (Table 1), describe its evolution into clinical practice, and highlight its many applications for congenital heart disease.

## 2. Evolution of CMR/MRA Contrast Agents

While non-contrast MRA methods have desirable attributes, MR signal enhancement through the use of pharmaceutical contrast agents can greatly improve the reliability and diagnostic power of CMR/MRA. The use of gadolinium-based contrast agents (GBCAs) in MR angiography has been in clinical use for over three decades. Although the diagnostic utility and safety of the GBCAs have been established [14,25], concerns over the association with nephrogenic systemic fibrosis (NSF) and gadolinium deposition in various tissues have spurred interest in alternative agents that do not contain gadolinium [26,27,28].

Gadolinium is a rare earth metal with strong paramagnetic properties, and it is the active species in virtually all MRI contrast agents used worldwide. The toxicity of isolated gadolinium salts is well established, such that all GBCAs are chelates and organic ligands prevent the dissociation of the free gadolinium ion [29]. The chelates are classified as linear or macrocyclic, depending on their structure and, as a general rule, the macrocyclic structure is more stable to dissociation. Initially, high doses of linear GBCAs were employed in MRA and in the mid-2000s, an association between GBCA exposure and the development of NSF was established. While the pathogenesis of NSF is poorly understood, one theory involves prolonged tissue retention of GBCA secondary to renal impairment resulting in release of free gadolinium and subsequent tissue deposition, leading to fibrosis. This association led to stricter guidelines on usage of GBCAs especially in neonates and adults with renal impairment [30]. Under the new guidelines, GBCA use was still considered safe with normal renal function, until evidence of gadolinium deposition in brain tissue of patients with normal renal function who had undergone multiple GBCA-enhanced MR exams [31]. Since these reports, subsequent animal studies have shown gadolinium deposition was more associated with linear agents compared to macrocyclic agents; illness severity and clinical signs, however, still vary from case to case [14,25].

As noted above, depending on the shape of the organic ligand, GBCAs are categorized into linear or macrocyclic. Older linear agents carry a higher risk of NSF and soft tissue deposition [32,33,34]. Currently there are nine GBCAs approved by the United States Food and Drug Administration for utilization in MRI and macrocyclic agents are preferred [29].

While gadolinium is not found in normal human tissue, iron is an essential element involved in various physiological functions. Approved for therapeutic use as an intravenous iron supplement, ferumoxytol (Feraheme, Covis Pharmaceuticals, Waltham, MA, USA; generic ferumoxytol, Sandoz Inc., Basel, Switzerland) has emerged as a promising alternative contrast agent [13,15,28]. Due to its long intravascular half-life of more than 15 h, ferumoxytol is considered a true blood pool agent [35]. The long steady state intravascular half-life provides opportunities for repeat scans without loss of image quality, as well as for more time-consuming sequences such as 4D MRA (MUSIC) and 4D flow [24]. The r1 relaxivity is also several-fold higher for ferumoxytol as compared to available GBCAs [26]. This allows for high vascular signal enhancement on T1-weighted imaging. Table 2 highlights the advantages and disadvantages of GBCAs and ferumotyxol.

## 3. Repurposing of Ferumoxytol for Off-Label Diagnostic Use and Its Safety

As noted above, iron is a substance critical for health, whereas gadolinium is foreign to biological systems. This has helped to spearhead the investigation of ferumoxytol as an alternative MR contrast agent, especially in light of recent concerns for gadolinium deposition in the brain and other tissues [28,36]. Ferumoxytol was approved by the FDA in 2009 as therapy for iron deficiency anemia in adults with chronic kidney disease. However, in 2015 it was linked to serious hypersensitivity reactions, including 18 deaths [28]. Pooled postmarketing adult clinical trial data for the therapeutic use of ferumoxytol at that time revealed the rate of acute anaphylaxis to be 0.03% [37]. By comparison, anaphylactic reactions with GBCAs can be up to 0.01% of cases [38]. It should be noted that the initial therapeutic use of ferumoxytol involved rapid injection of an entire vial (510 mg, 17 mL) over 17 s. The initial adverse effects related to therapeutic ferumoxytol administration may have been due to higher total iron dosage and fast bolus injection. For diagnostic purposes, ferumoxytol is diluted and administered at a slower rate of injection, and at lower doses than those given for therapy [39,40].

Since initial reports of serious hypersensitivity related to therapeutic use in 2015, several single-center safety studies on the off-label diagnostic use have emerged [17,40,41]. The results were positive. Subsequently, as an increasing number of centers began to trial this “off-label” use of ferumoxytol, the FeraSafe Consortium Registry was created. FeraSafe is a large multicenter MRI safety registry with the purpose of investigating the safety of ferumoxytol-enhanced MRI among patients with various clinical indications including CHD [19]. In that study, which included 4240 ferumoxytol injections in patients with ages ranging from 1 day to 96 years, no severe or fatal adverse events were reported. Mild or moderate reactions deemed related or possibly related to ferumoxytol injection occurred with 1.9% of the injections; none were reported in children.

Transient hypersensitivity reactions characterized by myalgias with headache and chest pressure have been described in association with intravenous iron infusions. This self-limiting event, known as a Fishbane reaction, does not usually recur with subsequent infusions [42]. It is important to distinguish this mild and self-limited reaction from true anaphylactoid and anaphylactic reactions. The treatment of a Fishbane reaction might be to simply stop the infusion and monitor the patient, while anaphylaxis requires immediate attention with administration of epinephrine along with potentially advanced airway and cardiovascular interventions.

An additional concern involves the possibility of iron overload with ferumoxytol injections. While serum iron levels could be tested when there are clinical concerns about hereditary conditions, iron-deficiency and anemia are much more common occurrences worldwide, especially in chronically ill patients [43].

Currently, the FDA continues to recommend patient-specific dosing, dilution, and slow infusion rates, along with careful monitoring of vital signs pre- and post-infusion [44]. While clinical trials have yet to be performed to directly investigate the safety of ferumoxytol use in a diagnostic setting, monitoring and assessment of adverse events have been promising thus far, laying the groundwork for full clinical development in the future [19].

## 4. Ferumoxytol-Enhanced MRA (FE-MRA) and 4D Phase Contrast MRI (4D Flow) in Congenital Heart Disease

Awareness of the distinct imaging advantages and favorable safety profile of ferumoxytol-enhanced MRA (FE-MRA) has led to its increasing use in children and adults with CHD. The challenges involved in imaging CHD include (1) complex intracardiac and extracardiac vascular anatomy, (2) requirement for high temporal and spatial resolution, (3) need for repeated examinations over a lifetime, (4) anesthesia in younger patients and some older patients, (5) and regional variability in CMR availability and/or CHD expertise. These, and other challenges, can potentially be addressed through the use of ferumoxytol [6,8,18,24,41,45].

In the following sections, we present clinical scenarios in which FE-MRA and 4D Flow have been useful for pediatric and adult CHD. Table 3 reviews the advantages of ferumoxytol over GBCAs in specific congenital heart conditions.

### 4.1. Pulmonary Venous Anatomy

#### 4.1.1. Total Anomalous Pulmonary Venous Connection (TAPVC)

TAPVC is a critical congenital heart lesion typically presenting in neonates with severe cyanosis, respiratory distress, and severe pulmonary hypertension. Historically, TAPVC carries high morbidity and mortality risk [46]. The pulmonary veins are present but do not connect normally to the posterior left atrium. Rather, they maintain their embryologic systemic venous connections, often through a “vertical vein” which directs oxygenated blood abnormally towards the right heart.

**Figure 1:**
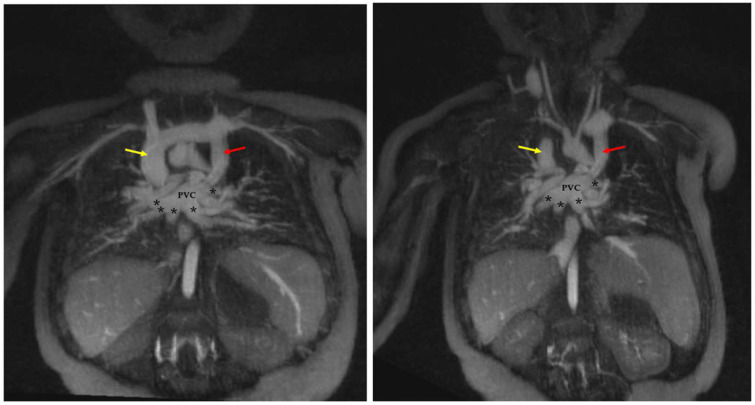
Total Anomalous Pulmonary Venous Connection (TAPVC), supracardiac type. In this neonate, coronal reconstructions from ferumoxytol-enhanced 4D MRA (MUSIC) clearly show abnormal connection of the pulmonary veins (black asterisks) connections to a pulmonary venous confluence (PVC), rather than the left atrium. Pulmonary venous blood then drains via superior “vertical veins” (red arrows), innominate vein, and then to the dilated right superior vena cava (yellow arrow).

Figure 1 depicts pre-operative assessment of a neonate with free-breathing time-resolved 4D FE-MRA of TAPVC (supracardiac type) with pulmonary venous blood draining abnormally via a superior “vertical vein” towards the superior vena cava. The additional findings of a midline liver and unbalanced atrioventricular canal defect (not shown) provided the additional diagnosis of heterotaxy syndrome. The patient underwent successful reconnection of the pulmonary veins to the posterior left atrium with ligation of the vertical vein.

#### 4.1.2. Pulmonary Vein Stenosis

**Figure 2:**
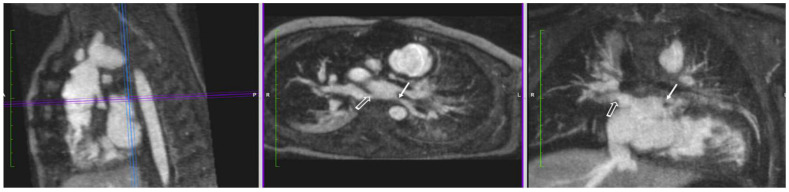
Pulmonary Vein Stenosis (acquired). Reformatted projections from 4D MUSIC images in an infant with hypoplastic left heart syndrome. Acquired postoperative stenosis of the left superior and inferior pulmonary veins after stage I surgical palliation is demonstrated (solid arrows). Narrowed right superior and inferior pulmonary vein ostia are also seen (open arrows).

Congenital or acquired stenosis of one or more of the pulmonary veins can be a serious condition with the risk of recurrent pulmonary infections and pulmonary hypertension [47]. Moreover, the size of the veins, their ostia, and the surrounding tissue may be difficult to image with echocardiography [48]. CMR/MRA can provide radiation-free and high-resolution images of these small structures. Additional information about individual pulmonary vein flow is also obtainable by 4D flow. An example of postoperatively acquired left superior and inferior pulmonary vein stenosis is shown in Figure 2.

### 4.2. Systemic Venous Anatomy

#### Total Cavopulmonary Anastomosis (Fontan Circulation)

**Figure 3:**
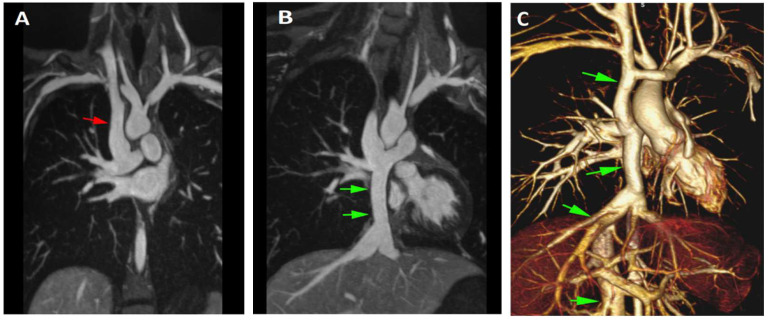
Fontan Circulation. Breath-held and ECG-gated FE-MRA in an adult patient with single ventricle heart disease who has undergone Fontan surgical palliation (i.e., routing of the SVC and IVC to the branch pulmonary arteries). Panel (**A**): Coronal maximum intensity projection depicting the superior limb (Glenn shunt) of the systemic venous drainage (red arrow). Panel (**B**): Coronal maximum intensity projection in a more posterior position from that in Panel (**A**) showing the inferior limb (Fontan conduit) of the systemic venous drainage and its connection to the right branch pulmonary artery (green arrows). Panel (**C**): 3D volume rendered image the image dataset which clearly and homogenously depicts the Fontan circulation (green arrows). The entire gated volumetric acquisition was acquired in a single breath-hold.

On the more complex end of the CHD spectrum are conditions with severe hypoplasia of one of the ventricles leading to “single ventricle” physiology. Most of these patients will undergo a staged surgical palliation strategy culminating in a total cavopulmonary anastomosis (i.e., Fontan surgery) used to reroute the systemic venous blood directly to the lungs and thus bypass the single ventricle. Slow transit of venous blood through these circuits makes bolus timing difficult in both MR and CT angiography. The requirement for precise timing, contrast dose, and injection parameters to provide adequate imaging of the cavopulmonary shunts involved in the Fontan circulation are eliminated by the use of a blood pool agent such as ferumoxytol (Figure 3) [49].

### 4.3. Arterial Pathology

#### 4.3.1. Coarctation of the Aorta

**Figure 4:**
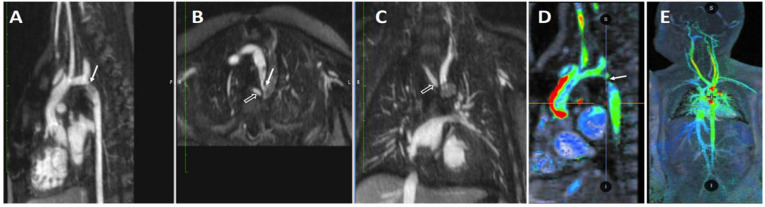
Coarctation of the Aorta. Panels (**A**–**C**): 4D FE-MRA (MUSIC) in a neonate with juxtaductal coarctation of the aorta (solid arrows) and hypoplastic transverse aortic arch. Additionally, present is an aberrant right subclavian artery (open arrow). Panels (**D**,**E**): Ferumoxytol-enhanced 4D flow in a different patient with coarctation showing anatomic narrowing of the aortic isthmus (solid arrow) with red flow (plus sign) indicating acceleration of blood flow across the area of stenosis.

An important cause of infant mortality, patients with critical coarctation of the aorta depend on timely diagnosis by echocardiography to avoid lack of systemic blood flow and cardiogenic shock. However, in postsurgical cases or in larger patients, acoustic imaging windows may be limited. CMR/MRA is the preferred advanced modality in these situations [50]. The free-breathing 4D FE-MRA technique adds further value with ability to image with higher spatial resolution and a better ability to discern maximum (systolic) dimensions of the aortic arch. The addition of a 4D flow sequence gives physiologic information about the severity of obstruction and collateral artery flow. The case of a neonate born with coarctation of the aorta and an aberrant right subclavian artery is shown in Figure 4.

#### 4.3.2. Aortic Aneurysm in Connective Tissue Disease

**Figure 5:**
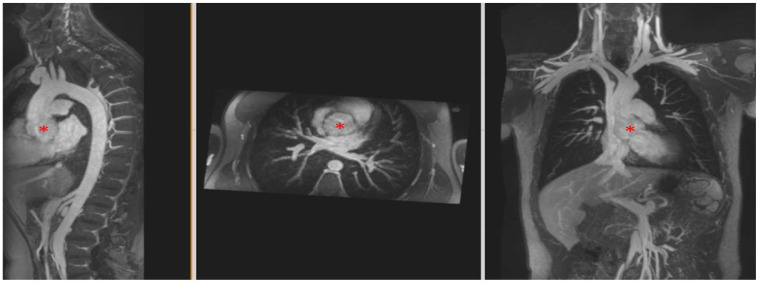
Aortic Root Aneurysm in Marfan Syndrome. Multiplanar reformatted breath-held FE-MRA (sagittal, axial, and coronal planes) in an adult patient with Marfan syndrome depicting an aneurysm of the aortic root (red asterisks). Additionally, homogeneous and high-resolution 3D images of the ascending, transverse (arch), descending thoracic, and upper abdominal aorta were possible in the same acquisition without the need for bolus timing.

Serial follow up of aortic dimensions is imperative in a variety of connective tissue disorders such as Marfan syndrome, Turner syndrome, bicuspid aortic valve, vascular Ehlers–Danlos syndrome, Takayasu arteritis, and Loeys–Dietz syndrome, to name a few [51]. Aortic imaging with CMR/MRA and CT angiography (CTA) is relatively straightforward in many centers. However, if imaging is not multiphasic, the systolic (largest) aortic dimensions may not be captured. Children pose additional challenges to imaging the aorta in terms of increased scan times (with prolonged exposure to anesthesia) to achieve the necessary spatial resolution, accurate ECG-gating at faster heart rates, and repeated scans over a lifetime [52]. While CTA is considerably faster than CMR/MRA, exposure to ionizing radiation in a condition requiring lifelong follow up may not be appropriate. On the other hand, conventional CMR/MRA in this patient population will require repeated doses of a GBCA with the concerns for gadolinium deposition, as discussed above. For these reasons, ECG-gated 4D FE-MRA may be the ideal modality for following patients with aortopathy. Comprehensive 3D imaging of all the segments of the thoracic and abdominal aorta, as well as the head and neck vessels, is achievable without GBCA or ionizing radiation exposure (Figure 5). In addition, when compared with standard CMR/MRA protocols, the duration of anesthesia may be markedly shortened using a ferumoxytol-enhanced 4D acquisition without breath-holding.

#### 4.3.3. Vascular Ring

**Figure 6:**
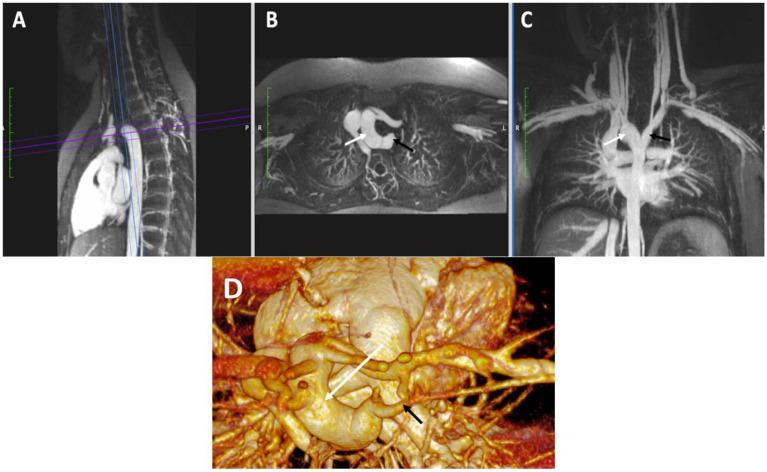
Vascular Ring. Reformatted maximum intensity projections from a breath-held FE-MRA study (Panels (**A**–**C**)) and 3D volume rendering (Panel (**D**)) depicting a right aortic arch (white arrows) with aberrant left subclavian artery (black arrows) arising from a diverticulum of Kommerell. This configuration forms a vascular ring causing mild tracheal compression in this patient.

Vascular rings are abnormalities of aortic arch development that can lead to external compression of the trachea and/or the esophagus. The most common forms of symptomatic vascular ring are: (1) right aortic arch with aberrant left subclavian artery arising from a diverticulum of Kommerell, and (2) double aortic arch. Clinical presentation ranges from asymptomatic, to stridor and/or wheezing, to dysphagia lusoria [53]. FE-MRA can help discern the specific types of rings and the time-resolved technique can yield information on dynamic compression of the trachea and esophagus (Figure 6). Although CT angiography has the advantage of superior airway image quality, its use of ionizing increased radiation is not desirable in this patient population, many of whom are newborns or young children at diagnosis.

#### 4.3.4. Pulmonary Atresia in Tetralogy of Fallot

**Figure 7:**
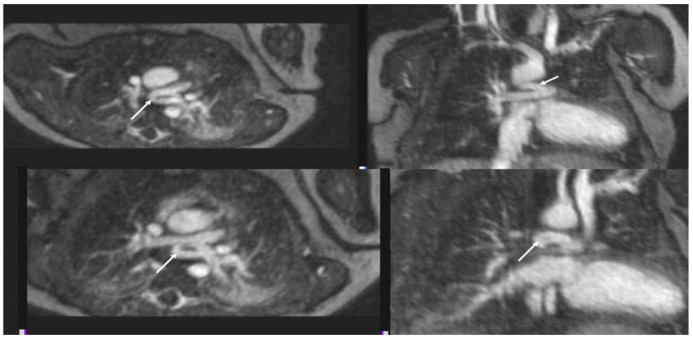
Tetralogy of Fallot with Pulmonary Atresia. Reformatted multiplanar maximum intensity projections from a 4D FE-MRA (MUSIC) study in a neonate. Continuous right and left branch pulmonary arteries are seen supplied by a patent ductus arteriosus (solid arrow) from the underside of the aortic arch. This study was used to plan transcatheter ductus arteriosus stent placement. No significant aortopulmonary collateral arteries were identified.

Abnormalities of the pulmonary arterial tree exist in several forms of complex CHD. Interference from the air-filled lungs can make imaging of the extracardiac vasculature by echocardiography challenging. CMR/MRA and CTA can overcome these limitations to generate high-quality 3D images of the proximal and distal pulmonary arteries. Planning transcatheter or surgical interventions in patients with conotruncal defects, for example, relies on this level of imaging [54]. Tetralogy of Fallot is a common type of conotruncal defect which can include atresia of the pulmonary vale and varying degrees of main and branch pulmonary artery stenosis. Steady-state imaging afforded by the use of ferumoxytol can facilitate MR visualization of both central and very distal pulmonary artery branches, as well as the presence and extent of aortopulmonary collateral arteries (Figure 7).

#### 4.3.5. Heterotaxy Syndrome and Double Outlet Right Ventricle

**Figure 8:**
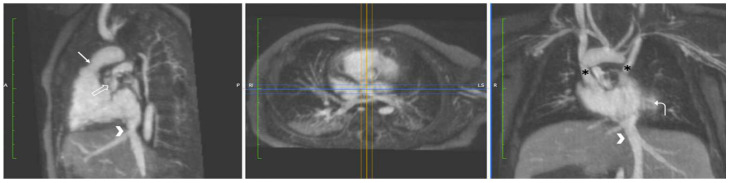
Double Outlet Right Ventricle with Pulmonary Stenosis (DORV/PS) in Heterotaxy Syndrome. Reformatted multiplanar maximum intensity projections from a 4D FE-MRA (MUSIC) study in an infant with DORV/PS. The aorta (solid arrow) arises from the right ventricle and is positioned anterior and rightward of the pulmonary artery (open arrow). The heart is positioned in the midline (mesocardia) with a leftward pointing apex (curved arrow). There are bilateral SVCs without a bridging vein (asterisks) and the IVC is left-sided, connecting to the leftward half of a common atrium (arrowheads). The liver is transverse.

Heterotaxy syndrome can coexist in patients with complex cyanotic CHD. These patients can have systemic venous connections that complicate surgical repair and must be delineated in advance. The case example presented is of an infant born with double outlet right ventricle and pulmonary stenosis in the setting of several systemic venous anomalies (Figure 8).

### 4.4. Intracardiac Anatomy

#### 4.4.1. Unbalanced Atrioventricular Canal Defect

Maldevelopment of the cardiac crux can lead to an atrioventricular canal defect, involving atrial and ventricular septal defects as well as failure of mitral and tricuspid valve separation (common atrioventricular valve). Typically, surgical closure of the septal defects and creation of separate atrioventricular valves corrects the condition. However, if ventricular inflow is directed preferentially towards the right ventricle (for example), the left ventricle may be underdeveloped at birth, and vice versa. A left ventricle too small to support the entire cardiac output may preclude the patient from a full surgical repair. As mentioned above, CMR/MRA has the ability to help adjudicate the adequacy of left ventricular size in these patients [55]. 

**Figure 9:**
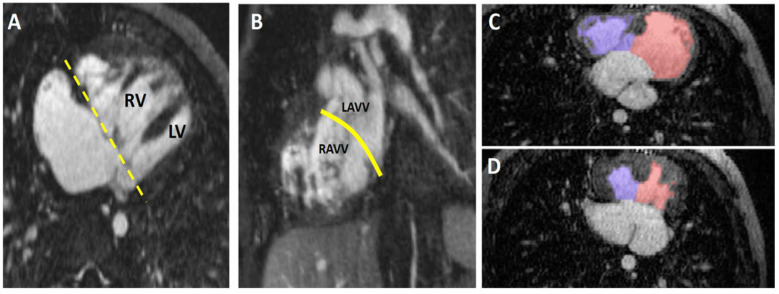
Unbalanced “Right-dominant” Atrioventricular Canal Defect (AVCD). Multiplanar maximum intensity projections from a 4D FE-MRA (MUSCI) study in an infant with unbalanced AVCD and relatively small left ventricle (“right-dominant”). This study was performed to determine candidacy for a full surgical repair. The left LV is significantly smaller than the RV (Panel (**A**)). The dashed yellow line in Panel (**A**) indicates the plane of the short-axis image shown in Panel (**B**). The plane indicating where the interventricular septum should be is depicted by solid yellow line in Panel (**B**). From this short-axis image the asymmetry of the right and left sides of the AV valve, and the relative paucity of LAVV tissue, can be appreciated. Panels (**C**,**D**) are images from a study in a different patient. LV (red shaded area) and RV (blue shaded area) volumes and ejection fractions were calculated from diastolic (Panel (**C**)) and systolic (Panel (**D**)) 3D image data taken during steady-state enhancement by ferumoxytol. RV = right ventricle; LV = left ventricle; AV = atrioventricular; LAVV = left atrioventricular valve.

A case of an infant with “right-dominant” unbalanced atrioventricular canal defect and left ventricular hypoplasia is presented in Figure 9. The use of 4D FE-MRA and 4D flow allowed for calculation of the left ventricular end-diastolic volume and aortic flow (left-sided cardiac output) calculations. High-resolution intracardiac anatomic imaging was also useful in judging the degree of asymmetry of valvar tissue and ventricular inflow across the common atrioventricular valve. Ultimately, these parameters were deemed not compatible with a complete surgical repair at time in this patient and the surgeon opted for a staged palliation approach with a bidirectional cavopulmonary (Glenn) shunt.

#### 4.4.2. Hypoplastic Left Heart Complex

Congenital hypoplasia of the left-sided cardiac structures exists on a spectrum of severity. On the extreme end is the hypoplastic left heart syndrome (HLHS), with various degrees of aortic and mitral valve stenosis or atresia, along with aortic arch hypoplasia and/or coarctation. On the milder end is the hypoplastic left heart complex (HLHC), involving small left-sided structures, but without significant stenosis or atresia of the chambers of valve tissue [56]. 

Patients with HLHS will be committed to a “single ventricle” surgical palliation strategy, whereas certain patients with HLHC may be candidates for the more favorable “biventricular” repair. As was reviewed in the case above, CMR/MRA has become crucial in the decision-making algorithm for such patients [55]. Preoperative CMR/MRA evaluation of these patients requires the following elements: (1) extracardiac aorta imaging with high spatial resolution, (2) intracardiac imaging of the valves and ventricles with cardiac-phase-resolved high spatial resolution, for the calculation of volumes and function, and (3) phase contrast data of the aorta and pulmonary outflows. This examination is typically only possible with sedation or general anesthesia. Covering all these elements with conventional CMR/MRA typically requires an exhaustive time-consuming protocol performed by specially trained technologists and involves numerous breath-held acquisitions. On the other hand, cardiac-phase-resolved 4D FE-MRA can condense this process to a single acquisition performed in minutes during uninterrupted ventilation. As outlined above, this approach provides excellent image quality and offers comprehensive evaluation for patients undergoing preoperative assessment of HLHC.

**Figure 10:**
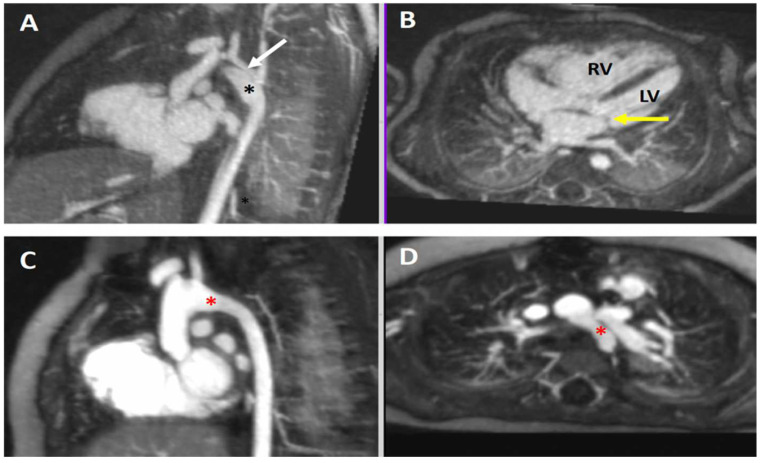
Hypoplastic Left Heart Complex (HLHC). Multiplanar maximum intensity reformatted projections from a 4D FE-MRA (MUSIC) study in a neonate with HLHC. Panels (**A**,**B**): There is a hypoplastic mitral valve (yellow arrow) and severe aortic arch hypoplasia with coarctation (white arrow). A large patent ductus arteriosus is also present (black asterisk). There is LV hypoplasia with relative enlargement of the RV. Panels (**C**,**D**): Postoperative images in the same patient using the same technique show a widely patient aortic arch after surgical repair (red asterisk). LV = left ventricle; RV = right ventricle.

Figure 10 depicts a case of HLHC in which the left ventricle was deemed by 4D FE-MRA to be of sufficient size to sustain biventricular circulation and avoid a single ventricle palliation. Thus, the surgery simply involved repair of the aortic arch. A widely patent aortic arch was seen on the follow-up study.

#### 4.4.3. Coronary Artery Anatomy

Anomalies of coronary artery anatomy occur not infrequently in CHD, with a prevalence of approximately 8% according to one study [57]. Precise knowledge of the origin and course of the coronary arteries is critical when planning for transcatheter or surgical therapies in CHD patients. 

**Figure 11:**
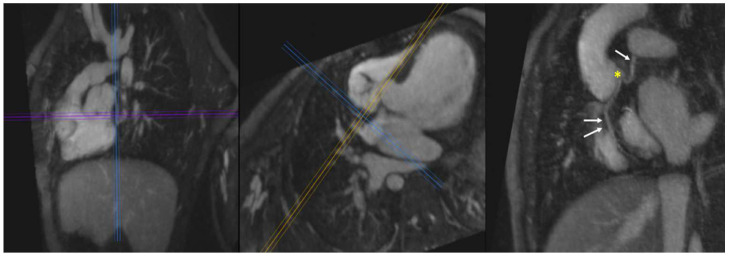
Single Coronary Artery. A single coronary artery is seen arising from the left aortic sinus in this young child with surgically repaired double outlet right ventricle and atrioventricular canal defect. Multiplanar maximum intensity projections from a 4D FE-MRA (MUSIC) study show the common origin of the right (double arrow) and left coronary (single arrow) arteries from the posterior aspect of the left aortic sinus (yellow asterisk).

In Figure 11, a case of double outlet right ventricle with single coronary artery is shown. In this patient, all coronary artery branches appear to arise from a single origin. Importantly, none of the coronary arteries in this case cross the right ventricular outflow tract. This is important to rule out prior to embarking on transcatheter or surgical procedures. 

**Figure 12:**
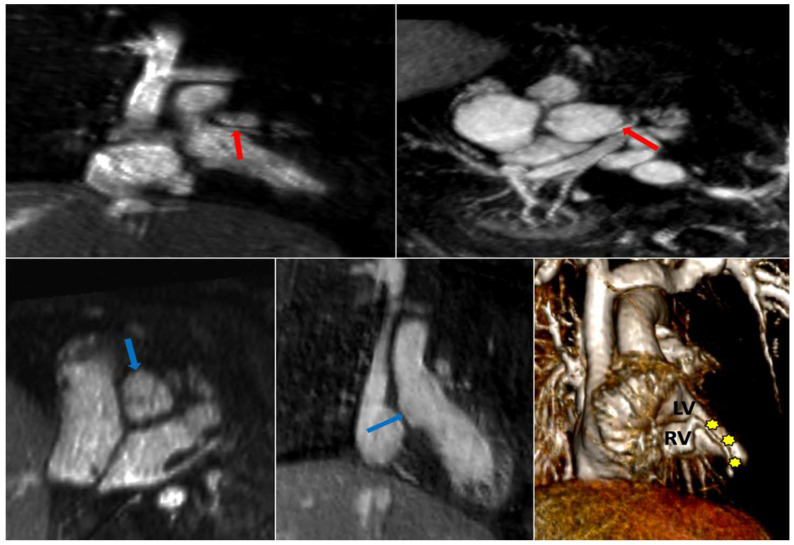
Absent Right Coronary Artery in Neonatal Pulmonary Atresia with Intact Ventricular Septum (PA-IVS). FE 4D MRA study (MUSIC) performed in a neonate on day 2 of life, reformatted in multiple planes, two showing normal origin of the left main coronary artery (red arrows), but right coronary ostial atresia (blue arrows). In the 3D volume rendered image, the right coronary artery (yellow stars) is seen arising directly from the right ventricular apex, consistent with right ventricle-dependent coronary circulation. Adapted with permission from reference [59]. RV = right ventricle; LV = left ventricle.

In pulmonary atresia with intact ventricular septum (PA-IVS), coronary artery anatomy ranges from normal to coronary ostial stenosis or atresia. Indeed, coronary artery branches can arise directly and completely from the body of the right ventricle. Thus, the choice of management algorithm hinges on accurate characterization of the coronary artery anatomy. Thus, PA-IVS patients frequently undergo invasive angiography with aortic root and direct RV injection [58]. In the case of PA-IVS presented, 4D FE-MRA done on day 2 of life confirmed suspicion of right coronary ostial atresia with origin of the right coronary directly from the apex of the RV (Figure 12). Given this finding, the patient was determined to be too high risk for catheterization and surgery and was directly referred for orthotopic heart transplantation.

### 4.5. Lymphatic Imaging

**Figure 13:**
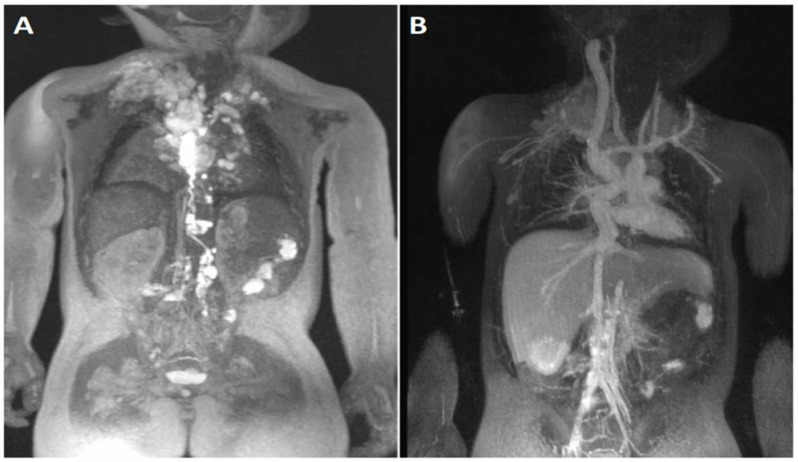
Combined MR Lymphangiography and FE-MRA. Panel (**A**): Coronal maximum intensity projection of a breath-held T2-weighted MR lymphangiogram performed after administration of dilute gadolinium via groin cannula. Panel (**B**): Coronal maximum intensity projection of a breath-held FE-MRA is also obtained to obtain the vascular anatomy and “overlay” the two images to guide transcatheter lymphatic therapy.

**Figure 14:**
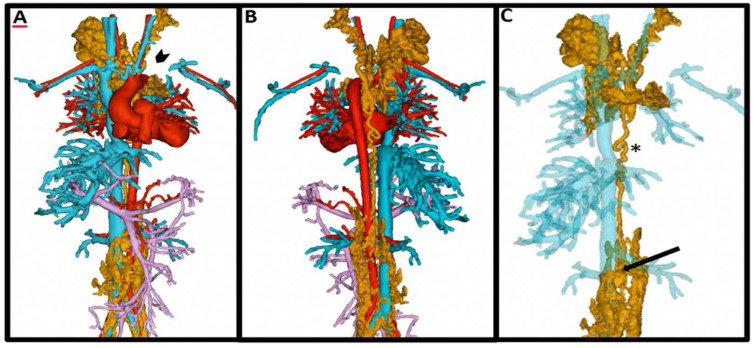
Ferumoxytol-enhanced 4D MRA (MUSIC) with overlayed dynamic contrast-enhanced MR lymphangiogram (DCMRL). Panels (**A**–**C**): Overlay of the lymphatic (gold), systemic venous (blue), arterial (red), and portal venous anatomy (lavender) arising from a dilated cisterna chylii (black arrow). In this patient with single ventricle anatomy and Fontan circulation, there is complete occlusion of the innominate, left internal jugular, and left brachiocephalic veins (arrowhead). The DCMRL shows a dilated and tortuous thoracic duct (*) with decompressing lymphoceles into the neck secondary to thoracic duct outlet obstruction. Figure courtesy of Dr. Sanjay P. Sinha, Dr. Arash Bedayat, and Takegawa Yoshida.

Anatomical and functional aberrations of the lymphatic system in these patients are seen at relatively high frequency as compared to patients with a biventricular circulation and are discernable with T2-weighted MR imaging [60]. Increased production and decreased reabsorption of lymph triggered by the higher central venous pressure inherent to the Fontan circulation can lead to debilitating and life-threatening complications such as protein losing enteropathy and plastic bronchitis. Precise mapping of the lymphatic system, as depicted in Figure 13 and Figure 14, has more recently helped to guide specific interventions for these conditions [61].

## 5. Future Applications

CMR/MRA, and ferumoxytol-enhanced CMR/MRA specifically, has an increasingly important role in the care of patients with CHD, potentially obviating the need for cardiac catheterization and/or CTA in certain patients. However, standard CHD imaging protocols in children can be complex, require specialized personnel and equipment, and are lengthy to perform [62]. The duration of the exam is especially an issue in the youngest patients who require anesthesia. However, at centers with specialized research agreements, ferumoxytol can be used with proprietary research pulse sequences to enable rapid acquisition of high-resolution, cardiac-phase-resolved 3D data with a requirement for only minimal technologist involvement. The addition of self-gating sequences and acceleration techniques, such as enhanced compressed sensing and artificial intelligence algorithms, may allow these examinations to approach the time efficiency of CTA [23,63,64]. Active collaborations with vendors are necessary to make these technical leaps more accessible at CHD centers that may not be attached to university hospital centers.

Currently, ventricular segmentation for ventricular size and function in CHD can be unwieldy when using 4D datasets. Much of the published data is derived from 2D analysis of multislice 2D cine images [59]. Although multiphasic CTA has provided a potentially higher fidelity method via volumetric analysis of the blood pool, its disadvantages lie primarily with the increased radiation dose associated with multiphasic acquisition, and to the challenges of proper bolus timing when both ventricles are being analyzed [65]. For these reasons, 4D CT acquisition is typically not performed in children. Recent work has shown that 4D datasets acquired using the multiphase steady-state imaging with contrast (MUSIC) pulse sequence can yield similar values for ventricular volumes and ejection fraction across both 2D and 3D software platforms (Figure 9, panels C and D) [66]. In the current state, volumetric and functional analyses are frequently manual or semi-automated, can be time-consuming, and require knowledge of CHD anatomy. Further development of machine learning algorithms to reduce the amount of labor associated with image segmentation will be important, particularly for CHD.

Coronary artery imaging by CMR/MRA has been utilized in the adult coronary artery disease population with success [67]. However, the smaller size of pediatric coronary arteries and the presence of coronary anatomic aberrancies make standard CMR/MRA protocols unreliable in this setting. The stable blood pool enhancement offered by ferumoxytol in steady state obviates the need for bolus timing. Due to speed and simplicity, coronary CTA and/or conventional angiography are often used in clinical practice for coronary evaluations in both adults and children. Our group has successfully applied cardiac phase-resolved 4D FE-MRA in very small babies to accurately delineate complex coronary anatomy (Figure 12) [68].

Despite the many uses of CMR/MRA in the CHD population, there remain important limitations in the youngest patients. In the current state, CTA can be acquired more rapidly, with high spatial resolution, and often without the need for anesthesia. However, 3D CT may not provide sufficient detail about intracardiac anatomy and blood flow, and in these areas, 4D MRI is powerful. The use of ferumoxytol with novel MRA techniques has opened up new vistas in non-invasive cardiovascular imaging of pediatric CHD at specialized institutions. With continued advancements in the speed of acquisition and the flexibility of image reconstruction, the hope is that these tools will become more widely available to the broader community, offering advanced imaging of CHD without radiation.

## 6. Conclusions

Diagnostic evaluation of congenital heart disease is a constantly evolving field seeking to deliver optimal image quality in the safest and most resource-efficient manner possible. Fortunately, concurrent developments in several imaging modalities have improved the quality of care delivered to this challenging patient population. CMR/MRA has an established track record of safe and comprehensive evaluation of the most complex congenital heart lesions in a wide range of ages and body sizes. The increasing adoption of ferumoxytol, along with the technical development of fast image acquisition and reconstruction methods, facilitates an ever-increasing role of MR imaging in CHD.

## Figures and Tables

**Table 1:** Select References Related to Off-Label Diagnostic Use of Ferumoxytol.

General MRI Contrast Agents (Ferumoxytol vs. Gadolinium)			
**Author/Year**	**Type of Article**	**Summary**	
Bashir et al., 2015 [13]	Review	Ferumoxytol shows potential to be used as a GBCA alternative in various applications of MRI, as well as in new in novel techniques due to its distribution within macrophages.	
Finn et al., 2017 [14]	Review	Ferumoxytol has several potential diagnostic applications and should be further investigated to define parameters for its safety and efficacy.	
Finn et al., 2020 [15]	Editorial	Obstacles to use of ferumoxytol for vascular imaging include: availability, expense, and off-label status.	
Daldrup-Link et al., 2022 [16]	Review	Ferumoxytol has long lasting blood pool enhancement and is useful in patients with renal insufficiency; however, it is contraindicated in patients with iron overload.	
**Safety**	**Population**	**Purpose**	**Outcomes**
Nguyen et al., 2017 *N* = 217 [17]	Patients (ages 3–94 years) at single center	Compare effects of ferumoxytol on monitored physiologic indices in patients under anesthesia with those of gadofosveset trisodium	No serious AEs with diagnostic use of ferumoxytol across wide spectrum of age, renal function, and indications.
Lai et al., 2017 N = 21 [18]	Neonates and young infants (1 day–11 weeks)	Evaluate feasibility of ferumoxytol-enhanced anesthesia-free cardiac MRI with rapid two-sequence protocol (4D flow and MRA) in complex CHD	One patient of 21 required additional imaging, one out of 13 with operative confirmation had a minor discrepancy. 4D flow was superior to MRA for evaluation of systemic arteries, valves, ventricular trabeculae, and overall quality.
Nguyen et al., 2019 N = 3215 [19]	Patients at 9 U.S. and 2 U.K. urban academic medical centers registered via FeraSafe multicenter MRI registry	Investigate incidence of acute adverse events for diagnostic ferumoxytol injection and describe registry practice pattern	No serious adverse events were recorded, minor infusion reactions were rare (<2%). Registry data revealed a lower rate of adverse events compared to post-marketing surveillance data for therapeutic use, correlating with different methods (lower total dose, slower average infusion rate, careful monitoring before/after).
**Congenital Heart Disease**	**Population**	**Purpose**	**Outcomes**
Ruangwattanapaisarn et al., 2015, *N* = 23 [20]	Pediatric patients (3 days–18 years)	Determine feasibility of ferumoxytol use in pediatric cardiac and vascular imagine (abdominal and cardiac MRA)	FE-MRA can achieve high image quality (high SNR) in abdominal cases and good blood pool to myocardium delineation for cardiac cases. Ferumoxytol dose of 1.5 or 3 mg Fe/kg were possible for venography
Zhou et al., 2017, *N* = 13 [21]	Pediatric CHD patients (4 days–13 years)	Validation of parallel imaging and compressed sensing combined reconstruction method for the 4D non-breath-held, multiphase, steady-state imaging technique (MUSIC)	CS-PI MUSIC reduced imaging time by approximately 50% while maintaining highly comparable image quality to the original MUSIC, with good reconstruction time (5 min).
Nguyen et al., 2017, *N* = 40 [22]	Pediatric patients (2 days–2 years)	Evaluate diagnostic performance and clinical value of 4D MUSIC in neonates/infants with CHD	FE-MUSIC provided accurate, high-quality images of cardiac and vascular anatomy. Findings on MUSIC, surgery, correlative imaging, and autopsy had excellent correspondence.
Han et al., 2017, *N* = 10 [23]	Pediatric patients with complex CHD (1 month–8 years)	Validate cardiac-respiratory self-gating (ROCK) strategy for multiphase steady-state imaging with MUSIC technique	ROCK-MUSIC provided equal or superior image quality and increased efficiency (40% scan time reduction) compared with original MUSIC.
Nguyen et al., 2021, *N* = 60 [24]	Pediatric patients, 20 each from 3 sites	Evaluate feasibility of 4D MUSIC MRI in pediatric CHD in a multicenter study	4D MUSIC MRI is feasible in a multicenter setting, reduces image acquisition time, and simplifies the acquisition protocol.

AE, adverse events; CHD, congenital heart disease; CS, compressed sensing; FE, ferumoxytol-enhanced; GBCA, gadolinium-based contrast agent; MRA, magnetic resonance angiography; MRI, magnetic resonance imaging; MUSIC, multiphase steady-state imaging with contrast; PI, parallel imaging; ROCK, rotating Cartesian k-space; SNR, signal-to-noise; 4D, 4-dimensional.

**Table 2:** Comparison of Gadolinium-Based Contrast Agents to Ferumoxytol.

Trait	Gadolinium	Ferumoxytol
Familiarity	Most commonly used MR contrast agents FDA-approved (including in children)	More recently employed as an MR contrast agent Off-label use for imaging purposes
Safety	Does not occur naturally in the body Very low rate of anaphylaxis	Iron is an essential element for physiologic function Very low rate of anaphylaxis Low incidence of mild, self-limiting, infusion reactions (especially in children, approximately 1–2%) Requires monitoring for 30 min after the infusion
Imaging Protocol	Requires precise bolus timing Separate arterial and venous phases Allows for myocardial perfusion and late enhancement imaging	No need for timing bolus Steady-state imaging Blood pool agent
Performance	High T1 relaxivity and signal-to-noise ratio Rapid blood transit	Superior T1 relaxivity and signal-to-noise ratio Stable blood concentration (supports high-resolution multidimensional imaging with uniform vessel signal)
Cost	In general, is less expensive than ferumoxytol preparations	Historically more expensive, but recent generic formulation has reduced the cost

**Table 3:** Advantages of Ferumoxytol over GBCAs in Specific Congenital Heart Lesions.

Diagnosis	Advantages of Ferumoxytol over GBCAs
Aortic Aneurysm	Genetic syndromes linked to aortic aneurysms and dissection include (among others): Marfan’s, Loeys–Dietz, vascular Ehlers–Danlos, and Turner’s. Bicuspid aortic valve also confers a higher risk. High-resolution imaging of the entire aorta and its branches can be readily achieved with FE steady-state imaging.
Congenital Coronary Anomalies	Imaging of these very small structures is facilitated with longer sequences focused on high spatial resolution. Also crucial to adequate coronary artery imaging in smaller patients is a bright and evenly enhanced blood pool, which is made more feasible using ferumoxytol.
Fontan circulation (for example, tricuspid atresia or HLHS)	The characteristic slow flow of the Fontan circulation can make precise MRA timing with GBCAs challenging given their relatively rapid vascular transit time. Both the superior limb (Glenn shunt) and the inferior limb (Fontan conduit), as well as the branch pulmonary arteries and collateral vessels, can be uniformly opacified during steady-state imaging.
Lymphatic Imaging	Overlay of FE MRA and contrast-enhanced MRL images has allowed high-resolution comprehensive mapping of the vascular tree as it relates to abnormal lymphatic connections in patients with conditions such as chylothorax, protein losing enteropathy, and plastic bronchitis. These imaging techniques have facilitated the development of novel transcatheter treatments.
TAPVC	FE steady-state imaging allows both the individual pulmonary veins and the abnormal systemic venous connection(s) to be visualized simultaneously in a single acquisition without the need for a timing bolus.
Tetralogy of Fallot	FE MRA can facilitate visualization of both the central and distal pulmonary artery branches, which are at risk for hypoplasia or atresia. In more severe forms of Tetralogy of Fallot, the pulmonary arteries can be replaced by aortopulmonary collateral arteries. Simultaneous visualization and precise mapping of these collaterals, and their relative contribution to regional lung perfusion as compared to the native pulmonary arteries, are critical to preoperative planning.

GBCA, gadolinium-based contrast agent; FE, ferumoxytol-enhanced; HLHS, hypoplastic left heart syndrome; MRA, magnetic resonance angiography; MRL, magnetic resonance lymphangiography; TAPVC, total anomalous pulmonary venous connection.

## Data Availability

Data reported in this study was extracted from published studies listed in the References. No original or newly analyzed data was provided in the manuscript.
